# Lifetime cognition and late midlife blood metabolites: findings from a British birth cohort

**DOI:** 10.1038/s41398-018-0253-0

**Published:** 2018-09-26

**Authors:** Petroula Proitsi, Diana Kuh, Andrew Wong, Jane Maddock, Rebecca Bendayan, Wahyu Wulaningsih, Rebecca Hardy, Marcus Richards

**Affiliations:** MRC Unit for Lifelong Health and Ageing at UCL, London, UK

## Abstract

Maintenance of healthy cognitive ageing is vital for independence and wellbeing in the older general population. We investigated the association between blood metabolites and cognitive function and decline. Participants from the MRC National Survey of Health and Development (NSHD, the British 1946 birth cohort) were studied; 233 nuclear magnetic resonance circulating metabolite measures were quantified in 909 men and women at ages 60–64. Short-term and delayed verbal memory and processing speed were concurrently assessed and these tests were repeated at age 69. Linear regression analyses tested associations between metabolites and cognitive function at ages 60–64, and changes in these measures by age 69, adjusting for childhood cognition, education, socio-economic status and lifestyle factors. In cross-sectional analyses, metabolite levels, particularly fatty acid composition and different lipid sub-classes, were associated with short-term verbal memory (4 measures in females and 11 measures in the whole sample), delayed verbal memory (2 measures in females) and processing speed (8 measures in males and 2 measures in the whole sample) (*p* < 0.002). One metabolite was associated with change in cognition in females. Most of the observed associations were attenuated after adjustment for childhood cognition and education. A life course perspective can improve the understanding of how peripheral metabolic processes underlie cognitive ageing.

## Introduction

Global increases in life expectancy have been accompanied by changes in labour market and social structures that place growing importance on the maintenance of healthy cognitive ageing for independence and wellbeing in the older general population^1^. It is therefore important to identify ways to maintain healthy cognitive ageing and to prevent or delay functionally significant cognitive decline, especially in the absence of effective disease-modifying treatments for dementia.

Blood metabolites closely represent the physiological status of an organism, reflecting what has been encoded by the genome and modified by systemic and environmental exposures^2^. Markers of lipid metabolism, such as essential fatty acids (FAs) and in particular omega-3 polyunsaturated FAs (n3-PUFAs), play a vital structural and functional role in the central nervous system, and are associated with cognitive performance and brain function during general ageing^3,4^. However, most studies linking lipids to cognitive ageing are limited by inability to control for potential reverse causality, since associations between lipids and cognition reflect lifetime interplay.

The MRC National Survey of Health and Development (NSHD, the British 1946 birth cohort) offers unique opportunities using an age-homogenous sample. Study members are now at an age where pathophysiological changes are likely to be accumulating, but frank dementia is still rare. A wide range of blood metabolites was assayed in late midlife (between ages 60 and 64) using nuclear magnetic resonance (NMR), and memory and processing speed were concurrently assessed. These cognitive tests were repeated at age 69. In addition, the NSHD has a wide range of potential confounders and mediators, including childhood cognitive ability^5^, education, lifetime socio-economic position, and health and health-related behaviours. We investigated blood metabolite levels in relation to cognitive function and decline.

## Materials and methods

### Participants

The Medical Research Council (MRC) National Survey of Health and Development (NSHD) is based on a nationally representative cohort of 5362 singleton births within marriage occurring during 1 week in March 1946 in England, Scotland and Wales. The cohort has been followed 24 times, most recently when participants were 68–69 years old^6^. Extensive information on sociodemographics, health and cognitive function has been obtained in childhood, adolescence and regularly thereafter^6^.

For the 60–64 years wave, 2229 of 2856 eligible participants (78.0%) underwent assessment. Contact was not attempted with those who were known to have died (*n* = 778), were living abroad (*n* = 570), had previously withdrawn from the study (*n* = 594) or were permanently untraced (*n* = 564)^7^. Of those assessed, 98% were willing to have a blood sample taken, and at least one blood sample was successfully obtained from 96%.

The participating sample remains broadly representative of native-born British men and women of the same age^7^. The current study protocol received ethical approval from the Greater Manchester Local Research Ethics Committee for the four English sites and from the Scotland A Research Ethics Committee. Written informed consent was obtained at each data collection.

### Metabolomics

Serum metabolomics analyses were performed on blood samples collected at ages 60–64. All blood samples were collected after an overnight fast and were not subjected to any free-thaw cycles prior to metabolomics. Serum metabolites were assayed using a high-throughput NMR metabolomics platform able to quantify up to 233 metabolite measures and ratios representing a broad molecular signature of systemic metabolism. Multiple metabolic pathways were covered, including lipoprotein lipids and lipid sub-classes, FAs and FA compositions, as well as amino acids and glycolysis precursors (Supplementary Table S1). Details are described elsewhere^8–10^. Following strict quality control (QC) serum metabolite data were available for 909 participants.

### Cognitive function

Cognitive function was assessed by short-term and delayed verbal memory, and speed of processing at age 60–64, and by change in these measures (except delayed verbal memory) by age 69. Short-term verbal memory was assessed by a three-trial 15-item word list learning task (maximum score = 45) devised by the NSHD^11^. After a processing speed task (see below), an uncued delayed free recall trial was administered (delayed verbal memory; 60–64 years only). Speed of processing was assessed as the number of letters P and W, randomly embedded within a page of other letters, crossed out as quickly and accurately as possible within 1 min (maximum 600)^11^.

### Covariables

The following variables were treated as potential confounders or mediators: sex, age at blood collection and blood collection centre, cognitive ability at 15 years, educational attainment and childhood and midlife SEP, BMI at 60–64 years^12^, lipid medication, lifetime smoking and alcohol consumption by 60–64 years, and exercise and nutrient intakes at 60–64 years^13–16^.

Cognitive ability at 15 years was represented as the sum of four tests of verbal and nonverbal ability^17^. Educational attainment by 26 years was grouped in three categories: no educational qualifications, ordinary (‘O’ level) secondary qualifications and advanced (‘A’ level) secondary or higher qualifications. Lifetime SEP was based on father’s occupation when study members were aged 11 (or if this was unknown at ages 4 or 15) and current or last own occupation at age 53; both were coded in six categories according to the UK Registrar General’s classification. Weight and standing height were measured at 60–64 years according to standard protocols, and BMI was calculated. Lipid medication was recorded by a research nurse as any lipid lowering drugs taken in the last 24 h before the blood sample was taken.

Lifetime smoking was represented by pack years per person from 20 to 64 years. Physical activity was defined as participating in any sports, exercises or vigorous leisure activities in the month preceding the age 60–64 interview (none, 1–4 times or more than 4 times). Participants recorded all alcoholic drinks consumed using 3–5 day diet diaries at 36, 43, 53 and 60–64 years^18^. An overall measure of adult alcohol consumption was calculated as the average of daily intakes (in grams) at all ages (when data were available for at least three of four waves); this was recoded as no consumption, light to moderate and heavy consumption across midlife.

Diet at 60–64 years was assessed using 5-day estimated diet diaries^19^. Mean daily consumption of the following nutrients were calculated when data was available for at least three days: total carbohydrates, total fat and total saturated FAs, total mono-unsaturated FAs, n3-PUFAs and n6-PUFAs, including any supplements taken; and nutrient densities per 1000 kcal were generated (grams/ total energy (kcal) × 1000).

### Statistical analyses

The outcomes of the study were the cognitive variables and the predictors were the metabolic measures. All cognitive variables were approximately normally distributed. Metabolite measures that showed any deviations from normality were transformed using the natural logarithm, or the natural logarithm plus 0.1 if they included zero values (Supplementary Table S1) n3-PUFAs and n6-PUFAs nutrient densities were skewed, so natural log transformed. Upon transformation all variables were approximately normally distributed. All outcomes and predictors were standardised for direct comparisons.

### Main analyses

**Fig. 1:**
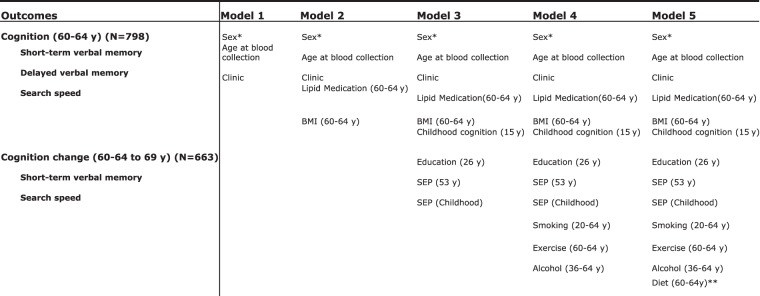
Stages of covariate adjustment. *Sex adjustment took place for whole-sample analyses. **Model 5 was run 6 times, using a different nutrient intake variable in each model (Table 1)

Linear regression was used to test associations between metabolites and memory and search speed. There were five stages of adjustments (Fig. 1): Model 1 adjusted for sex, age at blood collection and blood collection centre; Model 2 additionally adjusted for BMI and lipid medication at 60–64 years; Model 3 further adjusted for cognitive ability, educational attainment and lifetime SEP; Model 4 further adjusted for lifetime smoking, alcohol consumption and exercise at 60–64 years; Model 5 additionally adjusted for nutrient intake at 60–64 years; Model 5 was rerun 6 times including a different diet variable at each model. To maintain statistical power and minimise bias from missing data, we imputed missing values for the covariables in the sample of 798 participants with complete metabolite and cognition data at 60–64 years using multiple imputation chained equations (mice) implemented in R^20^. Sex interactions were tested in Model 1 and all models were sex-stratified when there was evidence of significant sex interaction (*p* < 0.1).

In the sample with a cognitive measure at age 69, we repeated this series of models for change (except delayed verbal memory) between age 60–64 and 69, by taking the difference between age 60–64 and 69 and adjusting for baseline (age 60–64).

To correct for multiple testing and correlation between metabolic measures, we set a metabolome-wide statistical significance threshold to *p* < 0.002 (Supplementary Figure S1); the *p* < 0.05 significance level was divided by the number of principal components^25^ that explained over 95% of variation in the metabolomics data. All analyses were conducted in R.3.1.1

### Additional analyses

In preliminary analyses we investigated associations between all covariables and the metabolites in the whole sample (adjusted for sex, age at blood collection and blood collection clinic) and in females and males separately (adjusted for age at blood collection and blood collection clinic) (Supplementary Table S2).

We performed sensitivity analyses by adjusting for one covariable at a time in addition to the Model 1 covariables (Supplementary Table S3 (a–c)). We also compared those with complete data through Model 3, to those with complete data on all covariates.

## Results

### Study sample characteristics

Of the 909 study participants with metabolite data, 798 had complete data at ages 60–64 for metabolite and cognitive measures. Of these, 663 participants had repeat cognitive measures at age 69. Characteristics of the samples are shown in Table 1.

**Table 1:** Characteristics of participants with complete data for all metabolites and cognitive measures at 60–64 years (*N* = 798) and both at 60–64 and 69 years (*N* = 663)

	Complete metabolite and cognition data for 60–64 y (*N*_max_ = 798)			Complete metabolite and cognition data for 60–64 and 69 y (*N*_max_ = 663^a^)		
	All	Women	Men	All	Women	Men
**SEP (Father’s),** ***n*** **(%)**	*N* = 768	*N* = 400	*N* = 427	*N* = 639	*N* = 312	*N* = 327
Professional		26 (6.9)	27 (6.9)		23 (7.4)	25 (7.6)
Intermediate		78 (20.7)	96 (24.6)		64 (20.5)	84 (25.7)
Nonmanual skilled		62 (16.5)	58 (15.5)		52 (16.6)	50 (15.3)
Manual skilled		120 (31.8)	121 (31.4)		102 (32.7)	102 (31.2)
Partly skilled		72 (19.1)	65 (16.6)		59 (18.9)	47 (14.4)
Unskilled		19 (5.0)	24 (6.1)		12 (3.9)	19 (5.8)
**Childhood cognition (15 y) (** ***z*** **-score), mean(SD)**	*N* = 705	*N* = 349	*N* = 356	*N* = 585	*N* = 288	*N* = 297
		−0.06 (0.96)	0.06 (1.03)		0.00 (0.93)	0.12 (1.02)
**Education (26 y),** ***n*** **(%)**	*N* = 760	*N* = 377	*N* = 383	*N* = 633	*N* = 314	*N* = 319
No qualification		112 (29.7)	118 (30.8)		91 (28.9)	88 (27.5)
Up to GCSE		126 (33.4)	74 (19.3)		103 (32.8)	62 (19.4)
A-level or higher		139 (36.9)	191 (48.9)		120 (38.2)	169 (53.0)
**SEP (15–53 y),** ***n*** **(%)**	*N* = 796	*N* = 390	*N* = 406	*N* = 662	*N* = 324	*N* = 338
Professional		6 (1.54)	58 (14.3)		5 (1.54)	50 (14.8)
Intermediate		164 (42.1)	180 (44.3)		145 (44.8)	155 (45.9)
Nonmanual skilled		128 (32.8)	39 (9.6)		105 (32.4)	35 (10.4)
Manual skilled		34 (8.7)	97 (23.9)		25 (7.7)	72 (21.3)
Partly skilled		46 (11.8)	23 (5.7)		34 (10.5)	19 (5.5)
Unskilled		12 (3.08)	9 (2.2)		10 (3.09)	7 (2.1)
**BMI (64** **y) (weight (kg)/height (m)**^**2**^**), mean(SD)**	*N* = 797	*N* = 389	*N* = 498	*N* = 662	*N* = 323	*N* = 339
		27.54 (4.7)	27.4 (3.9)		27.64 (4.8)	27.2 (3.8)
**Lipid Medication (64 y),** ***n*** **(%)**	*N* = 798	*N* = 390	*N* = 408	*N* = 663	*N* = 324	*N* = 339
Yes		68 (17.4)	105 (25.7)		63 (19.4)	86 (25.4)
No		322 (82.6)	322 (74.3)		261 (89.6)	253 (74.6)
**Physical activity (64 y),** ***n*** **(%)**	*N* = 769	*N* = 371	*N* = 398	*N* = 643	*N* = 312	*N* = 331
None		215 (58.0)	247 (62.1)		176 (56.4)	191 (57.7)
1–4 times a month		56 (15.0)	59 (14.8)		50 (16.0)	55 (16.6)
4 + times a month		100 (27.0)	92 (21.1)		86 (27.6)	85 (25.7)
**Lifetime smoking (20–64 y),** ***n*** **(%)**	*N* = 645	*N* = 310	*N* = 335	*N* = 534	*N* = 256	*N* = 278
Pack years per person		10.83 (15.3)	13.25 (17.5)		10.12 (15.2)	12.08 (16.9)
**Lifetime alcohol consumption (36–64 y),** ***n*** **(%)**	*N* = 668	*N* = 324	*N* = 344	*N* = 563	*N* = 270	*N* = 293
No consumption		27 (8.3)	13 (3.8)		22 (8.2)	9 (3.1)
Light-moderate consumption		277 (85.5)	252 (73.3)		229 (84.8)	220 (75.1)
Heavy consumption		20 (6.2)	79 (22.9)		19 (7.0)	64 (21.8)
**Diet (64** **y), mean daily nutrient densities/1000** **kcal, mean (SD)**	*N* = 680	*N* = 336	*N* = 344	*N* = 586	*N* = 283	*N* = 303
Carbohydrates		118.53 (18.1)	114.99 (18.7)		118.77 (18.2)	114.72 (18.8)
Fat		37.98 (6.7)	37.55 (6.2)		38.01 (6.9)	37.57 (6.2)
Total saturated FAs (FA)		14.36 (3.8)	14.09 (3.41)		14.31 (3.7)	14.08 (3.5)
Total mono-unsaturated FAs (MUFA)		12.53 (2.7)	12.74 (2.6)		12.54 (2.7)	12.72 (2.6)
n3-polyunsaturated FAs (n3-PUFA)		1.09 (0.4)	1.04 (0.4)		1.10 (0.4)	1.05 (0.4)
n6-polyunsaturated FAs (n6-PUFA)		5.74 (1.8)	5.41 (1.8)		5.75 (1.8)	5.40 (1.8)

^a^Out of the 135 participants who were not included in the analyses at age 69, 45 were not approached as they had died (*n* = 38) or had been lost to follow-up (*n* = 7). The remainder 90 were approached but temporarily refused to participate (*n* = 42), did not respond (*n* = 19), withdrew (*n* = 4) or did not have full assessment completed (*n* = 25)

### Cognition at 60–64 years

#### Short-term verbal memory

**Fig. 2:**
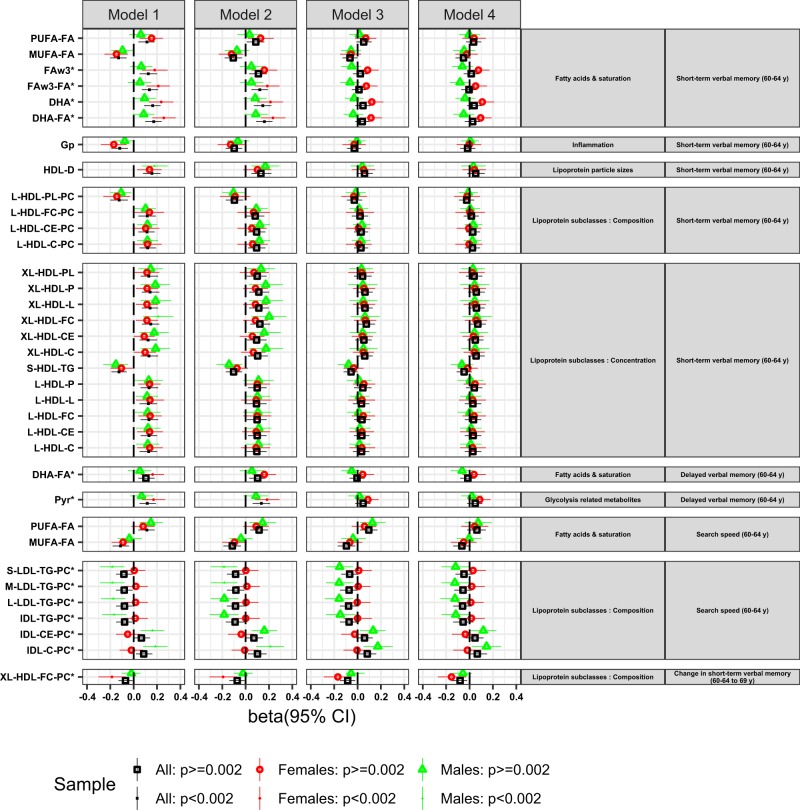
Linear regression results for the associations between metabolite measures and cognitive outcomes (short-term and delayed verbal memory and search speed at 60–64 years, and change in short-term verbal memory between 60–64 and 69 years) for Models 1–4. Only metabolite measures showing an association with the cognitive measure at metabolome significance threshold *p* < 0.002 in the whole sample or in sex-stratified analyses (when metabolite sex interaction *p* < 0.1) are presented. Association magnitudes are indicated in units of 1 SD metabolite concentration per 1 SD of each cognitive outcome. Coloured shapes indicate β-regression coefficients and the 95% confidence intervals. Each colour and shape represents the whole sample, or males and females. Filled shapes indicate associations passing metabolome significance threshold. The full names of the metabolite measures can be found in Supplementary Table S1. * indicates an interaction between metabolite and sex at *p* < 0.1

Of the eleven metabolite measures with significant sex modification (*p* < 0.1) in Model 1, four were associated with short-term verbal memory in females after correction for multiple testing (Fig. 2, Model 1): omega-3 Fas (FAw3) and DHA and their ratios to total FAs (FAw3-FA and DHA-FA respectively); the strongest association was with DHA-FA (beta = 0.256, 95% CI 0.16–0.36, *p* = 4.94 × 10^–7^). Adjustment for BMI and lipid medication slightly reduced some of these associations (Model 2) but most were attenuated by childhood cognitive ability, education and SEP, particularly by the first two (Model 3); there was no further attenuation after further adjustments for exercise, smoking and alcohol consumption (Model 4) and diet (Model 5).

Of the 222 metabolites with no significant sex modification, 11 were associated with short-term verbal memory (*p* < 0.002) in sex-adjusted analyses (Fig. 2, Model 1). The strongest association was with the diameter of high-density lipoproteins (HDL-D) (beta = 0.156, 95% CI 0.08–0.23, *p* = 4.11 × 10^–5^). The rest of the associations were mainly with lipids in large and X-large HDLs, with the ratio of poly and mono-unsaturated FAs to total Fas (PUFA-FA and MUFA-FA, respectively), as well as with glycoprotein acetyl (GP). The effects of adjustments in the whole-sample analyses were similar to those observed in females, with most associations being attenuated in Model 3.

### Delayed verbal memory

Two metabolite measures showed sex modification (*p* < 0.1) in Model 1. These two measures, pyruvate and DHA-FA, were associated with delayed verbal memory in females (Fig. 2, Model 1), the strongest association being with pyruvate (beta = 0.169, 95% CI 0.07–0.27, *p* = 1.00 × 10^–3^). These associations remained statistically significant in Model 2 but were attenuated in Model 3, and there was no further attenuation in Models 4 and 5.

None of the rest of the 231 metabolite measures were associated with delayed verbal memory in sex-adjusted whole-sample analyses.

### Search speed

Of the 40 metabolite measures with significant sex modification (*p* < 0.1) in Model 1, eight were associated with search speed in men. The strongest association was with total cholesterol in intermediate density lipoprotein (IDL-C) (beta = 0.187, 95% CI 0.08–0.29, *p* = 4.21 × 10^–4^), and the rest of the associations were with the ratios of triglycerides (TG), cholesterol and cholesterol esters (ChoE) to intermediate and low density lipoproteins (IDLs and LDLs). These associations remained, albeit weakened, after adjustments (*p* < 0.05, Model 5).

In whole-sample sex-adjusted analyses, MUFA-FA and PUFA-FA were associated with search speed (Fig. 2; Model 1). The strongest association was with MUFA-FA (beta = −0.113, 95% CI −0.18 to −0.04, *p* = 0.1.5 × 10^–3^). These associations were attenuated in Model 2 and further weakened by additional adjustments.

**Fig. 3:**
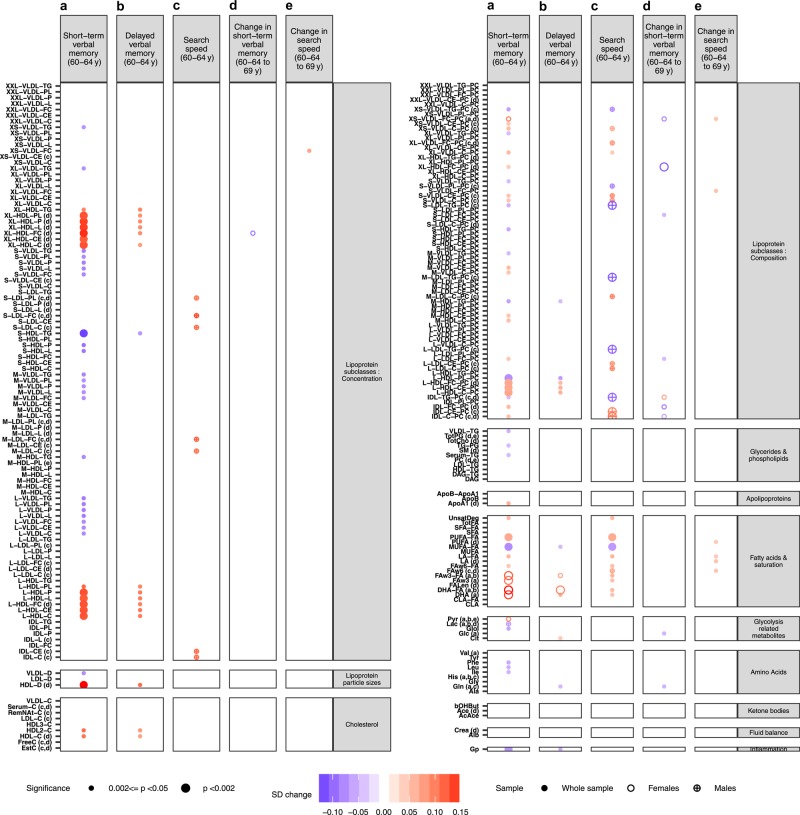
Linear regression analyses results for the associations between all metabolite measures and cognitive outcomes. **a** Short-term verbal memory, **b** delayed verbal memory and **c** search speed at 60–64 years; **d** change in short-term verbal memory and **e** change in search speed between 60–64 and 69 years for Model 1. Small circles represent associations at 0.002<=*p* < 0.05 and large circles at *p* < 0.002. Colours represent the association magnitudes are indicated in units of 1 SD metabolite concentration per 1 SD of each cognitive outcome. (**a**–**e)** An interaction between metabolite and sex at *p* < 0.1 for each outcome in which case sex-stratified analyses are performed for the respective outcome. Full circles represent sex-adjusted analyses performed in the whole sample and hollow and crossed circles represent analyses performed in females and males respectively in the case of an interaction with sex. The full names of the metabolite measures can be found in Supplementary Table S1

Figure 3 shows the associations between all metabolites and cognitive outcomes at *p* < 0.05; the cross-sectional associations between all metabolites and the cognitive outcomes are presented in Supplementary Table S4.

### Cognitive change between 60–64 and 69 years

Forty-three metabolites showed sex modification and one, the ratio of free cholesterol to total lipids in XL-HDL (XL-HDL-FC-PC), was associated with greater decline in short-term verbal memory in females (beta = −0.186, 95% CI −0.30 to −0.07, *p* = 1.31 × 10^–3^), with slight attenuation in Model 3. No associations were observed between the rest of the 232 metabolites and change in short-term verbal memory in whole-sample sex-adjusted analyses.

Of the remaining 190 metabolites, 4 showed evidence of sex modification but none were associated with change in search speed between 60–64 and 69 years in sex-stratified analyses. There were no associations between the rest of the metabolites and change in search speed between 60–64 and 69 in whole-sample sex-adjusted analyses.

Figure 3 shows the associations between metabolites and change in cognition at *p* < 0.05; the associations between all metabolites and change in cognition are presented in Supplementary Table S4.

## Discussion

Using a large British population-based birth cohort we investigated associations between 233 blood metabolites at 60–64 years, and memory and speed of processing at the same age, and change in these cognitive functions from 60–64 to 69 years. We observed associations between different metabolite classes, especially FA and lipoprotein sub-classes, and cognition, some of which were sex specific. These associations were independent of health and health-related behaviours but were largely explained by childhood cognition and education (Supplementary Table S2), particularly for PUFAs and HDLs.

This is to our knowledge the largest single study to systematically investigate how the midlife blood metabolome is associated with midlife cognition and cognitive decline, and how life course factors explain these associations. We used a representative British birth cohort study with metabolite data representing a broad molecular signature of systemic metabolism, and concurrent measures of memory and processing speed. Cognition was re-assessed at age 69, at a stage of the life course when pathophysiological changes relevant to CNS function are likely to be accumulating, but dementia is still rare. The main strength of our study is the range of potential confounders and mediators. These include rarely-available childhood cognitive ability, education, socio-economic status, BMI, lipid medication, diet information, exercise and lifetime smoking and alcohol consumption information. Building sequential models we were able to interrogate the influence of all these covariables on the associations of metabolites with cognition. Our study also has some limitations. These include lack of metabolite data at age 69, which precludes investigation of covariate changes in metabolite levels and cognition; and lack of childhood metabolite data, which disallows detailed investigation of life course bi-directionality between the metabolome and cognition. Further studies may investigate the latter using appropriate instrumental approaches such as Mendelian randomisation (MR).

With these strengths and limitations in mind, how should these findings be interpreted?

The adult human brain comprises about 20% of the whole body’s cholesterol^8^ and contains the largest diversity of lipid classes. n3-PUFAs may optimise the efficiency and plasticity of synaptic transmission in the brain; may dampen glial-activated pro-inflammatory events caused by stress; and may promote the renewal of neuronal cells in the hippocampus^21^, a key structure for normal and abnormal cognitive ageing. Studies have shown associations between cognitive ageing and AD and DHA containing phosphatidylcholines (PCs) in blood^3,22,23^ and brain tissue^24,25^, as well as between DHA and general cognitive ability and dementia^4^, with Mfsd2a identified as the transporter of DHA through the blood–brain barrier^26^. A number of studies have investigated the association of long chain PUFA (LC-PUFA) supplementation with AD and although results are overall inconsistent a recent meta-analysis of randomised control trials indicated that n3-PUFAs supplementation seems to have beneficial effects on systemic endothelial vasodilator function and cognitive function^27^. However, the concentration of essential FAs decreases in the ageing brain^28^. Mechanisms for this are uncertain, but likely include dietary changes, impaired desaturase activity, increased lipid peroxidation through impaired antioxidant systems, and impaired vascular health^28^. Here, we found positive associations with PUFAs and negative associations with MUFAs and cognition, with the associations of n3-PUFAs and DHA with cognition being observed only in women. This is consistent with previous work that has shown that the cognitive benefits of DHA were more profound in women^27^. Although it has been reported that such sex-specific associations could be attributed to bmi differences between the two genders^29^, the association of DHA and cognition in women here was independent to bmi. Future studies will interrogate the complex interplay between n3-PUFA, cognition and gender.

Our study also points to associations between cognition and different lipid subfractions. These included associations between short-term verbal memory and subfractions of Large and XL HDLs, including ChoEs, PCs, TGs and free cholesterol, and associations between search speed and LDLs/IDLs subfractions, mainly TGs, in men. In addition to lipid transport, HDL regulates vascular health via mediating vasorelaxation, inflammation and oxidative stress, and promotes endothelial cell survival and integrity^30^. Although studies are not always consistent, HDLs have been implicated in age related cognitive decline and AD (reviewed in ref. ^31^) and were recently associated with general cognitive ability and dementia and AD^4^. Additionally, complex sex-specific associations between LDLs and IDLs and cognitive decline in older adults and AD have been previously reported^32,33^. However, Mendelian randomisation studies have shown no evidence of a causal association between HDL-C or LDL-C and AD^34,35^. Nevertheless, the association of these compositionally and functionally diverse lipoprotein particles in relation to cognition and potentially AD is not well understood and warrants further investigation. Interestingly, it has been suggested that PUFAs can alter serum lipid profile;^36–39^ serum omega-3 and omega-6 PUFAs have been associated with higher serum levels of large HDLs and HDL diameter and serum MUFA concentration has been inversely associated with Large HDL particles and positively associated with LDL particles. Indeed, here, we observed the same patterns of associations between serum lipid profiles and FAs (Supplementary Figure S1), although directionality was not established.

Finally, we found negative associations between glycoprotein acetyls (mainly a1-acid glycoprotein) and short-term verbal memory in the whole sample, and positive associations between pyruvate and delayed verbal memory in females. With regard to glycoprotein acetyls, changes in the level of several glycoproteins have been observed in the hippocampus and inferior parietal lobe in human AD;^40^ some of these glycoproteins interact with neurofibrillary tangles, leading to speculation that changes in their glycosylation may be associated with the pathogenesis of this disease^40^. Additionally, A1-acid glycoprotein was previously found to be a strong predictor of 10-year mortality^41^ and was also recently negatively associated with general cognitive ability^4^. Little is known about human cognition in relation to to pyruvate levels, but the present findings are consistent with relevant animal studies. For example, rodent models of AD suggest apparent neuroprotective effects of pyruvate administration; mechanisms include protection against beta amyloid oligomer-induced neuronal cell death^42^, and (consistent with essential FAs) reduction of lipid peroxidation and oxidative stress^43^.

An important and consistent finding from this study is that associations between metabolites, in particular FAs, and cognitive function, in particular verbal memory, were largely explained by childhood cognition and educational attainment. Indeed, we found one example of a stronger association for childhood cognition than adult cognition (n3-PUFAs in men (Supplementary Table 2)). With regard to childhood cognition, this is positively associated with healthy dietary choice in NSHD, even after taking account of education and lifetime SEP^44^. However, health behaviours were not important explanatory variables in this study (Supplementary Tables S3a–c) even though they were associated with FAs (Supplementary Table S2). Alternatively, this association may also reflect lifetime bi-directionality between FAs and cognition, beginning with maternal FA intake, which prolongs the duration of pregnancy^45^ (leading to heavier birthweight, itself positively associated with cognitive development^46^). FAs then cross the placenta, and separately enter breast milk. Breastfeeding, in turn, is positively associated with cognitive development, even when confounding from maternal cognitive ability is controlled^47^. From this perspective, adjusting for childhood cognition could be regarded as over-adjustment rather than removing a source of confounding; however, incorporating the interplay between early growth, nutrition and cognitive development, and how these influence later metabolite status and cognitive function, is beyond the scope of this study.

A third possibility is that the link between childhood cognition and metabolites is underpinned by a common genetic cause or by a combination of genetic and dietary or sex influences. Variation in FA desaturase (FADS) 1/2 genes, which influence rate of n3-PUFAs and n6-PUFA synthesis, contributes to blood concentrations of FAs^48,49^, with heritability estimates accounting for 32–70% of FA variation^48,50,51^. Complex interactions between FADS genotypes and maternal and infant dietary intakes and LC-PUFAs concentrations have been described, with maternal genetic variation in FADS frequently associated with lower concentrations of LC-PUFAs in maternal and infant blood and in breast milk^52–54^. Additionally, the breastfeeding effects on childhood cognition have been reported to be modified by FADs genes^55–58^, although studies are not always consistent^59,60^. Finally, a recent MR study has reported the effects of FADS genotypes on cognition in 8–11 years old schoolchildren to be sex specific^61^.

## Conclusion

Findings from this study improve our understanding of the peripheral metabolic processes underlying cognitive ageing. Our study suggests that the levels of circulating metabolites in midlife, in particular FAs and different lipid sub-classes, are associated with midlife cognition, and that some of these associations are sex specific. The attenuation of these associations, after taking into consideration childhood cognition and education, suggests that the metabolic profile may be altered earlier in the life course, conferring lifetime vulnerability to poor cognition. This highlights how adding life course information helps our understanding of these associations, which could have been otherwise overestimated in midlife. As metabolites are potentially modifiable markers through diet and lifestyle, these findings could hold special value in cognitive ageing research, and may contribute to risk-reduction strategies for cognitive impairment and dementia.

## Electronic supplementary material

Supplemental legends

sup table 3c

sup fig 1

sup table 1

sup table 2

sup table 3a

sup table 3b

sup table 4
