# Supplementary material for: Lifetime cognition and late midlife blood metabolites: findings from a British birth cohort
Source: Transl Psychiatry. 2018 Sep 26;8:203. doi: 10.1038/s41398-018-0253-0 (PMC6158182; doi:10.1038/s41398-018-0253-0)
Supplement: Supplementary file 1 — Supplemental legends [file 41398_2018_253_MOESM1_ESM.docx]

**Supplementary Information**

**Supplementary Figure S1.** Pairwise correlations of metabolite measures. Metabolite measures are ordered by metabolite type.

**Supplementary Table S1.** Names, abbreviations, type, units and transformation of metabolite measures used.

**Supplementary Table S2.** Linear regression analyses results for the association of each covariable against each metabolite measure. All analyses are adjusted for sex, age at blood collection and blood collection centre in whole sample and for age at blood collection and blood collection centre in sex-stratified analyses. The maximum N for each analysis was used (see Table 1, ages 60-64). The full names of the metabolite measures can be found in Supplementary Table 1.

**Supplementary Table S3 (a-c).** Linear regression analyses results after adjusting Model 1 separately for one covariable at a time in a) Whole sample (N=798); b) Females (N=390) and c) Males (N=408).. Analyses are adjusted for sex, age at blood collection and blood collection centre in whole sample analyses and for age at blood collection and blood collection centre in sex-stratified analyses Association magnitudes are indicated in units of 1-SD metabolite concentration per 1-SD of each cognitive outcome. The full names of the metabolite measures can be found in Supplementary Table 1.

**Supplementary Table S4.**  Linear regression analyses results for the associations between metabolite measures and cognitive outcomes (short-term and delayed verbal memory and search speed at 60-64 years, and change in short-term verbal memory and search speed between 60-64 and 69 years) for Models 1-4. As diet did not attenuate any of the observed associations, results for diet (Model 5) are not presented here. Association magnitudes are indicated in units of 1-SD metabolite concentration per 1-SD of each cognitive outcome. The full names of the metabolite measures can be found in Supplementary Table 1.
