# Supplementary material for: Lifetime cognition and late midlife blood metabolites: findings from a British birth cohort
Source: Transl Psychiatry. 2018 Sep 26;8:203. doi: 10.1038/s41398-018-0253-0 (PMC6158182; doi:10.1038/s41398-018-0253-0)
Supplement: Supplementary file 3 — sup fig 1 [file 41398_2018_253_MOESM3_ESM.pdf]

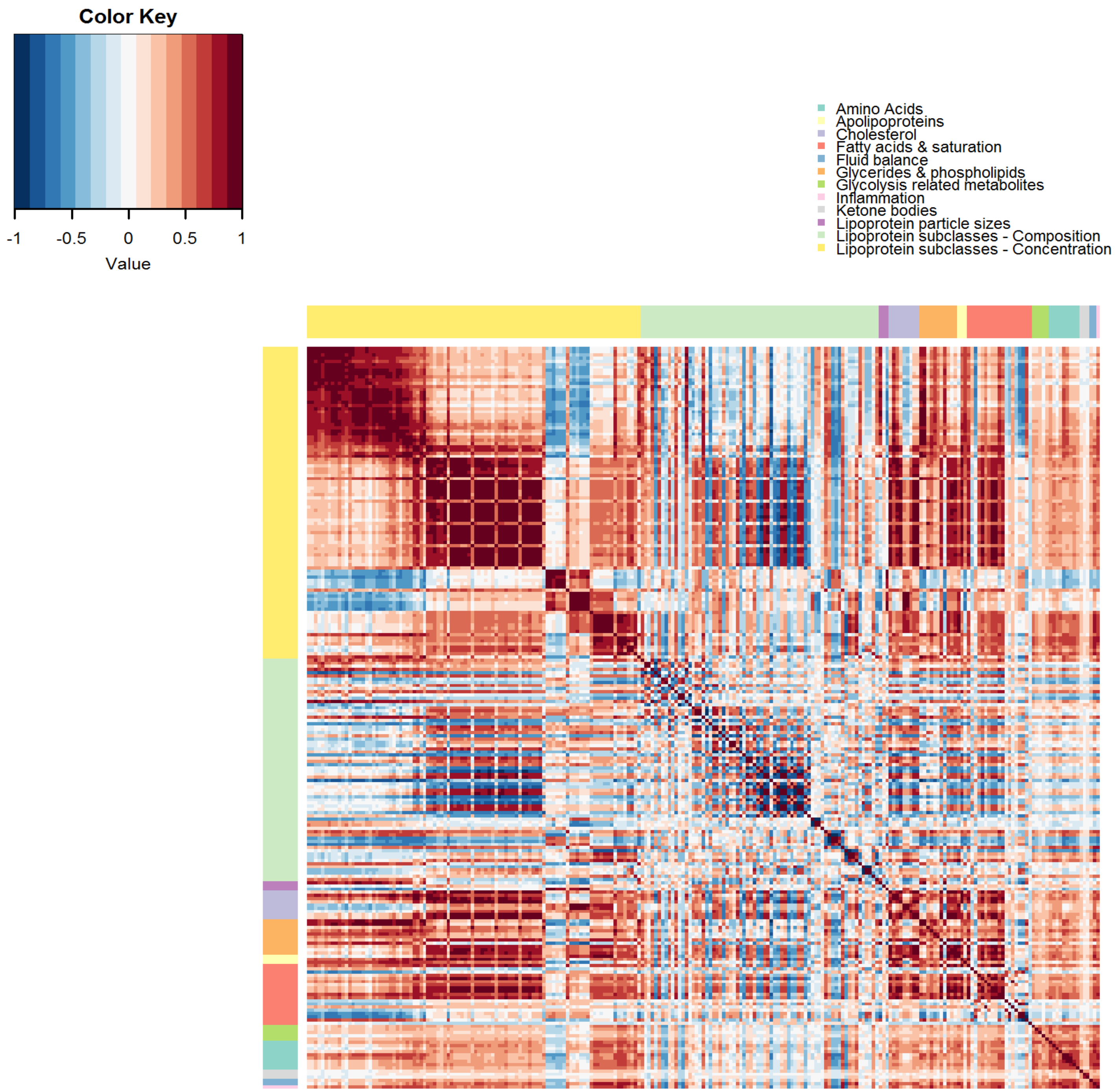

Color Key
-1
-0.5
0
0.5
1
Value
Amino Acids
Apolipoproteins
Cholesterol
Fatty acids & saturation
Fluid balance
Glycerides & phospholipids
Glycolysis related metabolites
Inflammation
Ketone bodies
Lipoprotein particle sizes
Lipoprotein subclasses - Composition
Lipoprotein subclasses - Concentration
